# Effect of Short Agonist Stop (SAS) Protocol in a Patient Having an Allergic Reaction From Conventional Ovarian Stimulating Hormone

**DOI:** 10.7759/cureus.54023

**Published:** 2024-02-11

**Authors:** Al Hera Ansari, Akash More, Shilpa Dutta, Namrata Choudhary, Jarul Shrivastava, Rokaiya Shaikh

**Affiliations:** 1 Clinical Embryology, Datta Meghe Institute of Higher Education and Research, Wardha, IND

**Keywords:** hmg injection, gnrh, lh, fsh, female infertility, allergic reaction, infertility

## Abstract

The case study examined a 32-year-old female and her 35-year-old partner who had experienced infertility attributed to the wife's irregular ovulation. Despite the male partner demonstrating normal reproductive parameters, he had a medical history involving hypertension. The woman also had a history of hypertension and chikungunya, which was accompanied by fever and joint pain in 2019. Due to the woman's allergic reaction to traditional ovulation stimulatory medications, the approach to assisted reproductive technologies (ART) had shifted towards employing the short agonist stop (SAS) regimen. Controlled ovarian stimulation had been achieved by administering recombinant follicular hormone, gonadotropin-releasing hormone (GnRH), and estrogen. The SAS protocol had been introduced to counter premature ovulation and synchronize follicular development. Following two successful ovum pickup procedures resulting in the retrieval of six embryos, the initial frozen embryo transfer took place in April 2022. Daily progesterone supplementation had been administered to sustain the patient's luteal phase. A subsequent human chorionic gonadotropin (β-hCG) test performed on the 14^th^ day post-embryo transfer confirmed a positive pregnancy diagnosis, with a measured outcome of 2026 mIU/mL. This case highlighted the potential impact of medication allergies on fertility outcomes and underscored the unique approach of the SAS protocol in managing allergic responses during in vitro fertilization (IVF) procedures. Despite the patient's history of allergic reactions to conventional ovulation stimulatory medications, the study concluded that the implementation of the brief agonist stop strategy had resulted in a successful clinical pregnancy.

## Introduction

Infertility is a condition defined as the incapacity of a couple to achieve conception after one year of unprotected intercourse [1]. It is a complex disorder that can manifest in both partners due to a multitude of contributing factors. When a woman demonstrates regular menstrual cycles and participates in unprotected sexual intercourse without achieving conception, this condition is classified as female infertility [1]. Reproductive system dysfunctions, hormonal imbalances, and a range of health-related complications may serve as underlying factors contributing to this condition [2]. Common aetiologies include ovulatory dysfunctions, fallopian tube obstructions, uterine abnormalities, endometriosis, hormonal fluctuations, fibroids or polyps, thyroid disorders, and lifestyle factors [3]. Primary infertility may arise from irregularities in ovulation, abnormalities in sperm, imbalances in hormonal regulation, age-related factors, and particular medical conditions [4]. Secondary infertility occurs when a woman successfully conceives at least once but faces challenges in subsequent attempts to conceive after a year of unprotected intercourse [5]. Causal factors encompass age-related changes, impaired sperm production, reproductive dysfunctions, hormonal imbalances, uterine anomalies, polycystic ovary syndrome, endometriosis, fluctuations in body weight, tobacco use, excessive alcohol consumption, and specific medication regimens. Strategies for enhancing fertility may involve pharmacological interventions employing assisted reproductive technologies (ART), including the administration of human menopausal gonadotropin (HMG) and follicle-stimulating hormone (FSH) [6]. These interventions play a crucial role in stimulating male sperm production and promoting ovarian follicle development in females [7]. In vitro fertilization (IVF) procedures integrate ovarian stimulation, utilizing protocols such as the short agonist stop (SAS), to modulate ovulation and promote the development of numerous follicles containing eggs, thereby enhancing the likelihood of successful conception [8]. Treatment modalities involve the administration of medications to women throughout each menstrual cycle to synchronize ovulation. The short agonist protocol is specifically designed to coordinate ovulation while simultaneously stimulating the development of ovarian follicles. This case report elucidates the impact of the SAS protocol on a patient experiencing an allergic reaction following the administration of HMG and FSH injections.

## Case presentation

Patient information

This case study was conducted at an Infertility Centre located in Wardha, Maharashtra, involving a patient who was 32 years old at the onset of treatment and her partner, aged 35 years. The patient presented with ovulatory dysfunction, posing a barrier to conception and resulting in secondary infertility, which prompted her to seek infertility treatment. Additionally, she had a documented episode of chikungunya infection in 2019, which was associated with thrombocytopenia. However, she exhibited complete recovery thereafter. Moreover, she had a medical history of hypertension, and there was also a familial predisposition to pre-eclampsia. The male partner and his relatives had no documented medical history, tobacco use, or alcohol abuse, although the male has a history of hypertension. It was determined from the semen analysis report of the male partner that he exhibited normal seminal parameters. Therefore, this indicates that the couple did not exhibit any male infertility factors. For a duration of five years, the couple encountered challenges with fertility subsequent to an eight-year marital union.

Consent was obtained or waived by all participants in this study and the Institutional Ethics Committee, Datta Meghe Institute of Higher Education and Research, issued approval DMIHER(DU)/IEC/2023/604.

Clinical findings

The patient presented with an abnormal ovarian reserve, as evidenced by the low level of anti-Müllerian hormone (AMH) of 0.23 ng/mL. The count of antral follicles was within the average range. Luteinizing hormone (LH) was quantified at 3.46 mIU/mL while FSH was measured at 4.43 mIU/mL. The patient experienced an allergic reaction following the administration of an ovulation stimulatory drug, HMG. Subsequently, we discontinued the medication midway through the controlled stimulation cycle.

**Figure 1:**
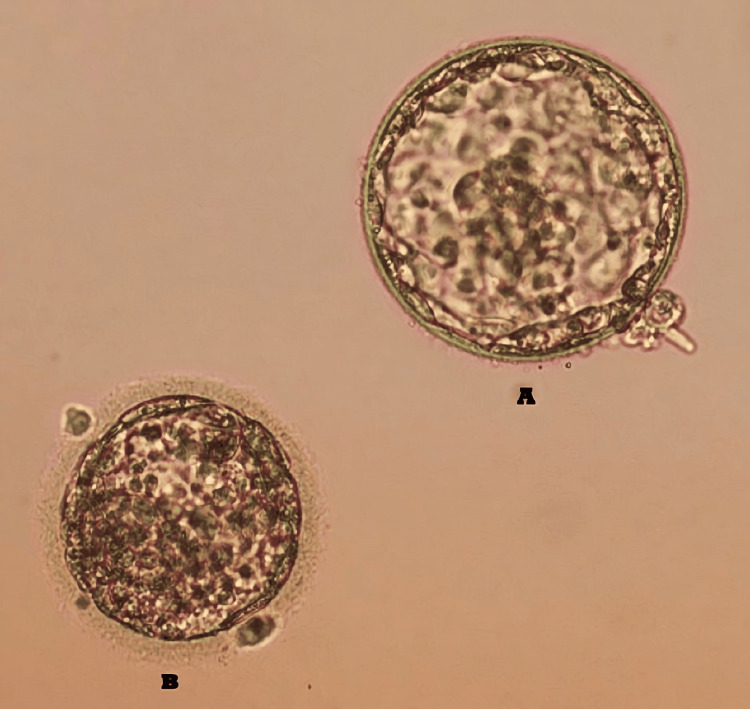
Blastocysts stage embryo of patient. A: 4BA embryo, B: 2BC embryo.

In the therapeutic intervention, we devised a protocol involving the administration of Cetrotide and gonadotropin-releasing hormone (GnRH) injections. The patient underwent agonist treatment spanning the initial to mid-follicular phase of the menstrual cycle. The administration of GnRH and Cetrotide injections modulates aberrations in the menstrual cycle and addresses ovulatory disorders. Nevertheless, a specific allergen sensitization protocol was devised for the patient due to an allergic reaction. On the first day, the patient underwent estrogen stimulation, and on the subsequent day, ultrasound was recommended concurrently with measurements of estrogen, luteinizing hormone, and progesterone levels. Commencing on the fourth day, administration of GnRH at a dosage of 0.1 mg was initiated and continued until the eighth day. Starting on day eight, the patient received guidance to undergo recombinant follicular hormone (RFH) supplementation. On the 14th day, the patient was triggered with a 10,000 IU dosage of human chorionic gonadotropin (β-hCG) hormones. Following a 36-hour interval post-trigger, oocyte retrieval procedure was performed. A subsequent procedure of follicular aspiration was performed to obtain mature oocytes. Eight oocytes in the M2 phase and two oocytes in the M1 phase were retrieved. After their formation, the six embryos underwent cryopreservation. On a daily basis, the patient was administered a 2.25 mg dose of Cetrotide along with GnRH injections. Progesterone tablets were utilized to support the luteal phase. On the fifth day, the patient underwent a subsequent embryo transfer cycle. Throughout the course of the treatment, no discernible complications were observed. Favourable outcomes were discerned through clinical monitoring conducted subsequent to the embryo transfer. Following a successful embryo transfer, the patient's uterine cavity harboured two embryos of high quality. Figure 1 shows the embryos that were transferred to the patient.

Follow-up

Furthermore, the patient received guidance regarding lifestyle modifications, encompassing recommendations for a balanced diet, consistent physical activity, and mitigation of potential risks. Regular follow-up appointments facilitated meticulous monitoring of the patient's progress and evaluation thereof. Diligent monitoring of the patient's overall health status and the progression of pregnancy heightened the likelihood of a favorable outcome. On the 14th day, the β-hCG test yielded a positive result at our Wardha laboratory, with a measured β-hCG value of 2026 mIU/mL.

## Discussion

The exogenous administration of human proteins has been associated with immunoglobulin (Ig) G-mediated reactions, such as antihuman LH antibody formation in a woman with isolated LH deficiency treated with human pituitary gonadotrophin for ovulation induction, resulting in secondary drug failure. Similarly, cases of antibody development against hCG post-ovulation induction have been reported. Studies in mice have highlighted the potential for allergic systemic IgE-mediated reactions to human menotrophins. Successful management of adverse local reactions to gonadotrophins has been achieved using highly purified FSH, with urofollitropin containing over 95% FSH. Eliminating non-hormonal proteins from urine-derived menotropins has reduced their allergenicity [9]. Ovarian stimulation is a pivotal element contributing to the efficacy of IVF and embryo transfer procedures by facilitating the recruitment of multiple viable oocytes suitable for fertilisation. Typically, ovarian stimulation involves the concurrent administration of GnRH analogues and gonadotropins, with the goal of averting premature luteinisation and fostering folliculogenesis, respectively [10]. Human follicle-stimulating hormone (hFSH), synthesized by the anterior pituitary gland, is a critical regulator of fertility across genders. Pharmaceutical formulations containing hFSH have been extensively employed for several decades in the management of infertility, particularly in women with anovulatory cycles and for controlled ovarian stimulation (COS) in individuals undergoing IVF. Furthermore, exogenous hFSH has been utilized in the therapeutic intervention of male hypogonadotropic hypogonadism [11]. Addressing poor responder patients poses a persistent challenge in the realm of assisted reproductive technologies. The SAS stimulation protocol employs a dual-phase stimulation approach involving initial activation with a gonadotropin-releasing hormone agonist (GnRH-a) followed by gonadotropin administration, coupled with a less stringent blockade (cessation of GnRH-a), to promote follicular recruitment and enhance ovarian response. This investigation compares the yield of oocytes attained through the SAS stimulation protocol against those obtained using the preceding stimulation regimen in the same cohort of women diagnosed with poor ovarian response (POR), as per the Patient-Oriented Strategies Encompassing Individualized Oocyte Number (POSEIDON) criteria [8]. Controlled ovarian hyperstimulation (COH) represents a pivotal stage in achieving favourable outcomes in vitro fertilization-embryo transfer (IVF-ET) procedures, facilitating the recruitment of multiple oocytes and subsequent vitrification of surplus embryos. Nevertheless, in certain patients characterized by extreme heterogeneity in ovarian response to COH, termed 'poor-responders,' the stimulation may result in minimal follicular development or none at all [12]. There are case reports available like Battaglia et al. which also reported allergic reactions to FSH during control ovarian stimulation. In line with the above studies, our patient also suffered from an allergic reaction upon the administration of HMG and FSH, which is regularly used in conventional stimulation IVF protocol. Hence, we advised the patient to undergo SAS protocol and it resulted in a positive clinical pregnancy in the patient. The concise short agonist cessation strategy is designed to synchronise follicular development and mitigate premature ovulation, thereby enhancing precision in regulating the duration of ovarian stimulation. This protocol distinguishes itself from others by employing GnRH agonists for a relatively brief duration, leading to a streamlined and expedited treatment cycle. The concise agonist cessation strategy represents an efficacious approach in reproductive medicine, endeavouring to enhance the rates of favourable outcomes in IVF cycles through optimization of the stimulation process [8].

## Conclusions

The implementation of a brief agonist cessation protocol led to the manifestation of positive clinical pregnancy outcomes in individuals experiencing allergic reactions to HMG and FSH injections. The attainment of a successful clinical pregnancy outcome through the brief agonist cessation protocol in a patient with prior allergic reactions to HMG and FSH injections underscores the versatility and efficacy of the protocol in addressing distinctive medical challenges. This underscores the significance of personalised approaches in ART, showcasing the capability to tailor protocols to individual patient needs, thereby improving outcomes in fertility treatment.
